# Apolipophorin-III Mediates Antiplasmodial Epithelial Responses in *Anopheles gambiae* (G3) Mosquitoes

**DOI:** 10.1371/journal.pone.0015410

**Published:** 2010-11-02

**Authors:** Lalita Gupta, Ju Young Noh, Yong Hun Jo, Seung Han Oh, Sanjeev Kumar, Mi Young Noh, Yong Seok Lee, Sung-Jae Cha, Sook Jae Seo, Iksoo Kim, Yeon Soo Han, Carolina Barillas-Mury

**Affiliations:** 1 Mosquito Immunity and Vector Competence Unit, Laboratory of Malaria and Vector Research, National Institute of Allergy and Infectious Diseases, National Institutes of Health, Rockville, Maryland, United States of America; 2 Department of Agricultural Biology, College of Agriculture and Life Science, Chonnam National University, Gwangju, South Korea; 3 Department of Parasitology, College of Medicine and Frontier Inje Research for Science and Technology, Inje University, Busan, Korea; 4 Johns Hopkins School of Public Health, Department of Molecular Microbiology and Immunology and Malaria Research Institute, Baltimore, Maryland, United States of America; 5 Division of Applied Life Science, Gyeongsang National University, Jinju, Korea; Charité-University Medicine Berlin, Germany

## Abstract

**Background:**

Apolipophorin-III (ApoLp-III) is known to play an important role in lipid transport and innate immunity in lepidopteran insects. However, there is no evidence of involvement of ApoLp-IIIs in the immune responses of dipteran insects such as *Drosophila* and mosquitoes.

**Methodology/Principal Findings:**

We report the molecular and functional characterization of *An. gambiae* apolipophorin-III (AgApoLp-III). Mosquito ApoLp-IIIs have diverged extensively from those of lepidopteran insects; however, the predicted tertiary structure of *AgApoLp-III* is similar to that of *Manduca sexta* (tobacco hornworm). We found that AgApoLp-III mRNA expression is strongly induced in the midgut of *An. gambiae* (G3 strain) mosquitoes in response to *Plasmodium berghei* infection. Furthermore, immunofluorescence stainings revealed that high levels of AgApoLp-III protein accumulate in the cytoplasm of *Plasmodium*-invaded cells and AgApoLp-III silencing increases the intensity of *P. berghei* infection by five fold.

**Conclusion:**

There are broad differences in the midgut epithelial responses to *Plasmodium* invasion between *An. gambiae* strains. In the G3 strain of *An. gambiae* AgApoLp-III participates in midgut epithelial defense responses that limit *Plasmodium* infection.

## Introduction

Lipophorins are protein-lipid complexes that transport lipid in the aqueous environment of the insect hemolymph [1], [2]. Three different apolipoproteins, apolipophorin-1 (apoLp-I, ∼250 kDa), apolipophorin-II (apoLp-II, ∼70–80 kDa), and apolipophorin-III (apoLp-III, ∼18 kDa), are present in insect lipophorins [2]. ApoLp-I and apoLp-II are generated by proteolytic cleavage of a common precursor protein called preproapolipophorin [3], [4] and form the “non-exchangeable” lipid-binding protein core of the lipophorin particle [2]. High-density lipophorin (HDLp) transforms into low-density lipophorin (LDLp) as it takes up diacylglycerol (DAG) and the lipid content of the particle increases. ApoLp-III is an “exchangeable” lipid-free hemolymph protein that changes conformation reversibly, as it binds to the LDL surface to prevent lipids in the particle from coming into contact with the aqueous environment [2]. The structure-function relationships of apoLp-IIIs have been extensively studied in *Manduca sexta* (tobacco hornworm) and *Locusta migratoria* (locust) model systems both *in vitro* and *in vivo* [5]–[8].

Besides its participation in lipid transport, apoLp-III has also been reported to mediate insect immune responses in several species such as *Galleria mellonella* (wax moth) [9]–[18], *Hyphantria cunea* (fall webworm) [19], *Heliothis virescens* (tobacco budworm) [20], and *L. migratoria* (locust) [21]. In *G. mellonella*, apoLp-III interacts with several immune elicitors such as lipopolysaccharide [16], lipoteichoic acid [14], and beta-1,3-glucan [17], suggesting that apoLp-III may act as a pattern recognition or as a carrier molecule. In addition, apoLp-III has been reported to increase antibacterial activity [9], [12], [17], [18], to be involved in activation of the prophenoloxidase cascade [17], and to participate in cellular immune responses [9]. For example, ApoLp-III increases the phagocytic activity of isolated hemocytes *in vitro* [9] and stimulates cellular encapsulation of foreign material [14]. Recently, it has been shown that apoLp-III protein is present in the granules of various insect hemocytes such as *H. cunea* prohemocytes, plasmatocytes, granulocytes, and spherulocytes [19]. In *M. sexta* [22], apoLp-III expression is induced in the late pupal stage, at a time when programmed cell death is taking place.

Lipophorin has been biochemically characterized in the malaria vector *Anopheles gambiae* and shown to be involved in lipid transfer to the developing eggs [23]. ApoLp-I/ApoLp-II mRNA levels increased in the midgut of *An. gambiae* (Yaoundé strain) mosquitoes infected with *P. falciparum*, and ApoLp-I/II silencing significantly reduced the oocyst density in mosquitoes infected with either *P. falciparum* or *Plasmodium berghei* [24]. In contrast, ApoLp-III midgut expression was not induced in response to *P. berghei* infection in the *An. gambiae* Yaoundé strain and ApoLp-III silencing did not affect the intensity of infection [24]. We characterized ApoLp-III (AgApoLp-III) in *An. gambiae* (G3) and explored the participation of AgApoLp-III in mosquito antiplasmodial responses. In G3 females *P. berghei* ookinete invasion triggers a strong transcriptional activation of AgApoLp-III in the midgut, and knockdown of this gene greatly enhances *Plasmodium* infection.

## Materials and Methods

### Mosquitoes

Experiments were carried out with the *An. gambiae* G3 strain. Larvae were grown in tap water after evaporating chlorine chemicals for two days and fed on TetraMin® Baby fish food (Spectrum Brands, Inc., Madison, WI) [25]. Adult mosquitoes were fed on cotton containing 10% sugar solution, blood fed on anesthetized mice (BALB/c), and kept in a 12:12-hour light:dark cycle at 26±2°C and 80% relative humidity.

### Infection of Mosquitoes with *P. berghei*

Female mosquitoes were infected by feeding them on *P. berghei*–infected BALB/c mice. Mouse parasitemias were determined, and potential infectivity to mosquitoes was established using exflagellation assays as previously described [26]. In all studies, mosquitoes were infected using mice with two to three exflagellations/field under a 40× objective. Blood-fed mosquitoes were kept at 21°C and 80% humidity. *P. berghei* infections were performed using a transgenic GFP-*P. berghei* strain (GFP-CON transgenic 259cl2 strain) [27] and the number of oocysts per midgut determined seven to eight days post infection. The distribution of the number of parasites per midgut in different experimental groups was compared using the Kolmogorov-Smirnov test, and all phenotypes were confirmed in three independent experiments. Public Health Service Animal Welfare Assurance #A4149-01 guidelines were followed according to the National Institutes of Health Animal (NIH) Office of Animal Care and Use (OACU). These studies were done according to the NIH animal study protocol (ASP) approved by the NIH Animal Care and User Committee (ACUC), with approval ID ASP-LMVR5.

### Identification, Sequence Analysis, and Phylogenetic Tree Construction

**Figure 1:**
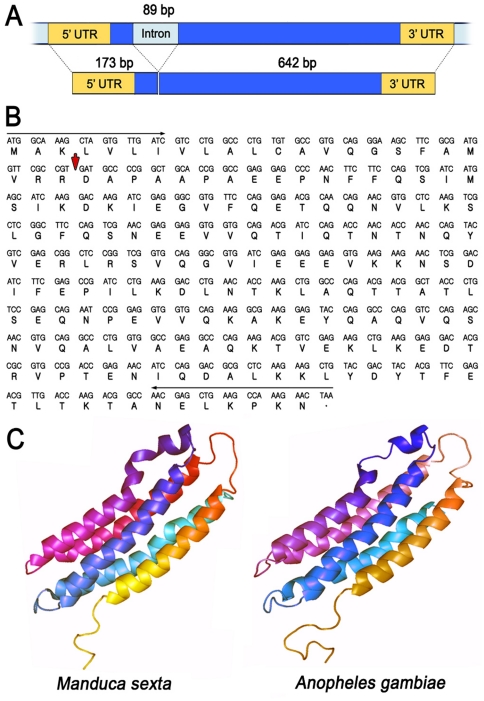
Genomic organization, cDNA and deduced amino acid sequence, and predicted tertiary structure of AgApoLp-III. (A) genomic organization, mRNA splicing and (B) cDNA sequence and deduced amino acid sequence of AgApoLp-III. (C) Tertiary structure of *Manduca sexta* and predicted structure of *Anopheles gambiae* ApoLp-IIIs.

The *Spodoptera litura* ApoLp-III cDNA sequence was chosen to identify a putative AgApoLp-III cDNA from the Ag genome database using the NCBI basic local alignment search tool (BLAST). Putative AgApoLp-III EST sequences were identified and used to design specific primers to amplify, clone, and sequence the cDNA. The sequence of the cloned AgApoLp-III cDNA (Figure 1B) was used to further investigate the genomic structure of AgApoLp-III by using the BLASTN program. Sequences of apoLp-IIIs from other species were obtained from NCBI, aligned, and an amino acid (aa) identity table built using Clustal-X2 (version 2.0.11) software [28]. Phylogenetic analysis was conducted using the neighbor-joining method [29] with bootstrap test (1,000 replicates) using the MEGA program (version 4.0.2) [30].

### Molecular Modeling of AgApoLp-III

The deduced amino acid sequence of *An. gambiae* ApoLp-III was submitted for automated protein structure homology modeling using the swissmodel protein fold server (http://swissmodel.expasy.org). The protein sequence alignment and three-dimensional coordinates of the known tertiary structure of *M. sexta* ApoLp-III were used as reference (PDB ID: 1EQ1).

### Sample Collection for RNA Isolation

Groups of ten mosquito samples were collected at different developmental stages: fourth-instar larvae, early pupae (pre-tanning stage), late pupae (tanned stage), and adult mosquitoes (male and females). Specific tissues were obtained by dissecting adult female mosquitoes under a stereomicroscope, immersed in *RNALater* solution (Qiagen, Chatsworth, CA) and stored at −80°C. For *Plasmodium*-infected samples, four- to five-day-old mosquitoes were fed on *P. berghei*-infected mice with five to ten exflagellations per field (40×) and kept at 20°C [25]. After 24 hours, 20 mosquitoes fed on a *P. berghei*-infected mouse or an uninfected mouse (control) was dissected at 20, 24, or 28 hours post infection (hpi). Midguts were dissected and stored separately from the remaining body parts (carcass) and stored at −80°C.

### RNA Isolation, cDNA Synthesis, and RT-PCR

Total RNAs were isolated from *An. gambiae* whole body using RNeasy Mini Kit (Qiagen) according to the manufacturer's instructions. Total RNAs were also isolated from the samples stored at −80°C, and first-strand cDNAs were synthesized from 5 µg of total RNAs using *AccuPower* RT PreMix (Bioneer, Daejeon, Korea) and an oligo (dT)_12–18_ primer as described in the manufacturer's protocol. The ribosomal protein S7 gene was used as positive internal control [31]. The numbers of PCR cycles for each target sequence were empirically adjusted to attain comparable band intensities in each experiment while avoiding saturation. The primers used were as follows: for S7A, 5′-GGCGATCATCATCTACGT-3′ S7B, 5'-GTAGCTGCTGCAAACTTCGG-3′; for *An. gambiae* apoLp-III A, 5′-ATGGCAAAG CTAGTGTTGATC-3′; for *An. gambiae* apoLp-III B, 5′-GTTCTTTGGCTTCAGCTCGTT-3′.

### Effect of Antibiotics on AgApoLp-III Expression in the Midgut

Two-day-old mosquitoes were fed for three days on 10% sugar solution containing penicillin (100 units/ml) and streptomycin (0.1 mg/ml) (Sigma Aldrich, St. Louis, MO) and then blood fed on either an uninfected or a *Plasmodium*-infected mouse. Midguts were dissected 20, 24, and 28 hours post blood feeding and AgApoLp-III mRNA levels determined by real-time PCR. Expression is presented as the fold induction relative to the expression levels at 20 hours in females fed an uninfected meal. S7 mRNA was used as an internal control to normalize the samples [31].

### Expression of AgApoLp-III in the Presence or Absence of Ookinetes

Females fed on an uninfected or infected mouse were kept either at 20°C (permissive temperature for ookinete development) or 28°C (non-permissive temperature). Midguts were dissected 24 hours post feeding and ApoIII mRNA levels determined by real-time PCR. Expression is presented as the fold induction relative to the expression levels in females fed an uninfected meal and kept at 20°C. S7 mRNA was used as an internal control to normalize the samples.

### Effect of Actinomycin-D-Induced Apoptosis on AgApoLp-III Expression

Mosquito females were fed on BSA alone or with BSA containing 10 µg/ml of actinomycin-D, a treatment known to induce apoptosis [32]. Midguts were dissected eight hours later and AgApoLp-III mRNA levels determined by real-time PCR. Expression levels were normalized using S7 mRNA as an internal control. Expression of *An. gambiae* dual oxidase (AgDuox, AGAP009978-PA), a gene known to be induced by actinomycin-D [32], was included as a positive control.

### Cloning AgApoLp-III into pRSET Expression Vector

*Kpn*I or *EcoR*I restriction sites were introduced at either end of *AgApoLp-III*-specific primers that generated a 579-bp PCR product (their position is indicated by the red arrow in Figure 1B). The PCR product was purified using a gel extraction kit (General Biosystem, Seoul, South Korea) after agarose gel electrophoresis and cloned into a TA vector (Invitrogen, San Diego, CA). The insert was released by digesting the plasmid with *Kpn*I and *EcoR*I, purified, ligated into the pRSET expression vector (Invitrogen), and used to transform DH5α cells. Three independent clones were sequenced to confirm that the cDNA insert was in frame.

### Expression and Isolation of Recombinant AgApoLp-III

A construct in the correct translation frame was used to transform BL21 cells and express recombinant AgApoLp-III protein. Overnight *Escherichia coli* cultures were diluted 1∶50 with LB media and grown at 37°C to 0.5 OD_600_. IPTG was added to a final concentration of 2 mM, and the cells were collected by centrifugation for four hours at 4°C. Most of the recombinant protein was found in inclusion bodies and was insoluble. Recombinant AgApoLp-III protein was partially purified following two-step procedures involving acid solubilization and SDS gel electrophoresis. The pellet containing the inclusion bodies was resuspended in 10% acetic acid, sonicated (ten times/ten seconds on ice) and centrifuged for 30 minutes at 13,000 rpm. The supernatant containing the solubilized recombinant protein was collected and evaporated using a Speed-Vac centrifugation system. The recombinant AgApoLp-III protein preparation was subjected to Tricine-SDS gel (16.5%) electrophoresis, and the induced protein band was cut from the gel after Coomassie blue staining. The gel band was homogenized in Dulbecco's phosphate buffer (Sigma) and gel slurry used for immunization.

### Immunization with Recombinant AgApoLp-III Proteins and Antibody Production

Gel slurry containing AgApoLp-III (1 ml) was mixed with the same volume of a complete adjuvant (Sigma) and injected subcutaneously into a rat. Two boost injections were administered every three weeks using gel slurry suspended in incomplete adjuvant (Sigma). The specificity of the anti-AgApoLp-III antibody was determined by western blot analysis.

### Western Blot

Homogenates from *An. gambiae* pupae were subjected to SDS-PAGE gel electrophoresis and blotted onto Immobilon-P polyvinylidene fluoride (PVDF) membranes (Millipore Corp., Bedford, MA). To reduce nonspecific background, PVDF membranes were incubated overnight in 10% BSA solution in TBS (10 mM Tris-HCl, pH 7.5, 100 mM NaCl) at 4°C. Subsequently, the membranes were incubated for three hours at room temperature (RT) with the primary (AgApoLp-III) antibody (1∶5,000) in TBS (10 mM Tris-HCl, pH 7.5, 100 mM NaCl) containing 5% (w/v) BSA. Membranes were washed three times for ten minutes with T-TBS (TBS containing 2.5% (w/v) BSA and 0.01% (v/v) Tween 20), and incubated with anti-rat IgG conjugated with goat alkaline phosphatase (Calbiochem, San Diego, CA) diluted 1∶2000 in 5% (w/v) BSA in TBS. Membranes were washed twice for ten minutes in T-TBS, and signals were developed by incubating the membranes with BCIP/NBT substrates in alkaline phosphatase buffer (Sigma).

### Midgut Immunostaining and Confocal Microscopic Analysis

Female adult *An. gambiae* infected with *P. berghei* were dissected 24 hpi. Midguts were isolated in PBS and fixed for one minute in 4% paraformaldehyde. The midgut contents were removed and the open midgut epithelia fixed for one hour at RT as previously described [25]. The midgut samples were permeabilized, and nonspecific antibody binding was blocked by incubating them for two hours in PBT (PBS with 1% BSA and 0.1% Triton X-100) at RT. Then the midgut samples were incubated overnight with anti-AgApoLp-III (1∶300) and anti-Pb28 antibodies diluted in PBT at 4°C. The midguts were washed three times with PBT for 20 minutes each and incubated for three hours at RT with the secondary antibody (1∶300) (Alexa 488-conjugated or Alexa 546-conjugated; Molecular Probes, Eugene, OR). The washing step was repeated as before: the midgut samples were mounted, and images were obtained using an Olympus FlowView 500 confocal microscope (Olympus Optical, Tokyo, Japan).

### dsRNA Synthesis

A 218-bp fragment of the lacZ gene was amplified using the primers (5′ to 3′) F-GAG TCAGTGAGCGAGGAAGC and R-TATCCGCTCACAATTCCACA and cloned into the pCRII-TOPO vector. For *AgApoLp-III*, a 297-bp fragment was amplified using the primers (5′ to 3′’) F-GCCGAGGAGCCCAACTTCTT and R-CACCACCTCCGGATTCTGCTC and cloned into the pCRII- TOPO vector. T7 promoters were incorporated onto ends of this fragment by amplifying the cloned insert using the following primers: M13F-CTCGAGTAATACGACTCA CTATAGGGCTAGTAACGGCCGCCAGTGT and M13R-CTCGAGTAATACGACTCACTA TAGGGGCCAGTGTGATGGATATCTGC. The PCR product was used as a template to synthesize dsRNA in vitro using the MEGAscript RNAi kit (Ambion, Austin, TX). dsRNA was further purified with water and concentrated to 3 µg/ml using a Microcon YM-100 filter (Millipore, Billerica, MA).

### Gene Silencing in Adult Female Mosquitoes

Female mosquitoes were injected with 69 nl of a 3 µg/ml solution of dsRNA of ApoIII at one to two days post emergence. Control mosquitoes were injected with dsLacZ. Four days later, females were fed on a *P. berghei*-infected mouse. Phenotypes were confirmed in three independent experiments. The silencing efficiency of AgApoLp-III in sugar-fed females, relative to controls injected with dsLacZ (100% expression), was 78%–90%.

## Results

### Gene Structure and Predicted Protein Folding of *AgApoLp-III*

The *AgApoLp-III* gene is located in the 2R chromosome of *An. gambiae* (33,372,097-33,373,233) (http://www.ensembl.org). The AgApoLp-III cDNA sequence was assembled based on the expressed sequence tags available in the database and reveals a transcript composed of two exons (173 bp and 642 bp) separated by one 89-bp intron (shown in light blue; Figure 1A). The AgApo-III cDNA is 815 bp long with a 579-bp protein-coding region (shown in blue) and 128-bp and 108-bp putative 5′ and 3′ untranslated regions (shown in yellow), respectively. The protein-coding region was amplified using the primer sequences shown in Figure 1B and sequenced (NCBI accession No. HQ112291). The predicted amino acid sequence codes for polypeptide of 193 aa, including a 23-aa putative signal sequence for secretion (MAKLVLIVLALCAVQ GSFAMVRR) (Figure 1B). The predicted cleavage site between amino acids VRR and DA, as proposed by Yamauchi et al. [33], is indicated by the red arrow and generates a mature AgApoLp-III peptide of 170 aa (∼19 kDa) (Figure 1B). Although the amino acid sequences of dipteran and lepidopteran ApoLp-III are highly divergent (see below) and only share about 20% identity, when we modeled the tertiary structure of *An. gambiae* ApoLp-III, the predicted structure is remarkably similar (Figure 1C) to that of *M. sexta* (PDB ID:1EQ1) [34], consisting of five long alpha-helices connected by short loops.

**Figure 2:**
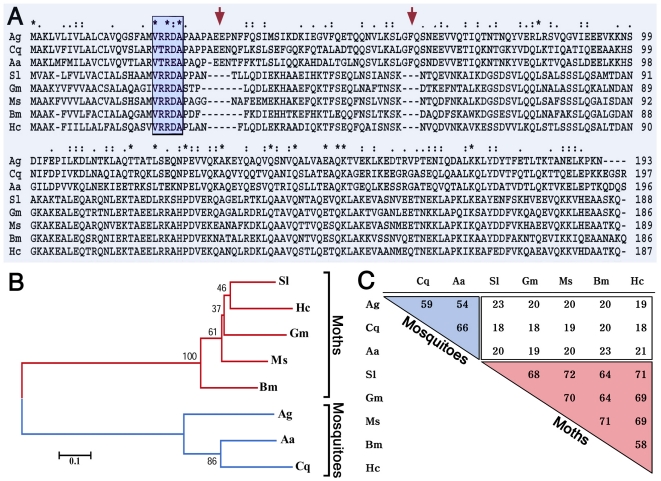
Sequence alignment, phylogeny, and sequence homology of insect members of the ApoLP-III family. (A) Sequence alignment of ApoLp-III sequences from *Anopheles gambiae* (Ag), *Culex quinquefasciatus* (Cq), *Aedes albopictus* (Aa), *Spodoptera litura* (Sl), *Galleria mellonella* (Gm), *Manduca sexta* (Ms), *Bombyx mori* (Bm), and *Hyphantria cunea* (Hc). Blue box (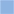) indicates highly conserved region; red arrows (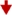) denote insertions in the sequence of mosquito members of the gene family. (B) Phylogenetic tree based on sequence alignment of the deduced amino acid sequence of members of the ApoLp-III family from different insect species. Mosquito sequences are indicated in blue and those from moths in red. (C) Sequence identity among ApoLp-IIIs from insects. The blue triangle (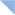) indicates identities among mosquitoes and the red triangle (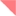) among moths. Notice the low level of sequence conservation between mosquitoes and moths (white area).

The sequence alignment of AgApoLp-III with other insect apoLp-III proteins revealed a high degree of conservation in amino acids 21 to 25 (VRRDA), which flank the signal peptide cleavage site (blue box; Figure 2A). There are two short insertions in mosquito apoLp-IIIs at positions 30–34 (ASEEP) and 62–64 (GFQ) that are not present in Lepidopteran apoLp-IIIs (red arrows; Figure 2A).

**Figure 3:**
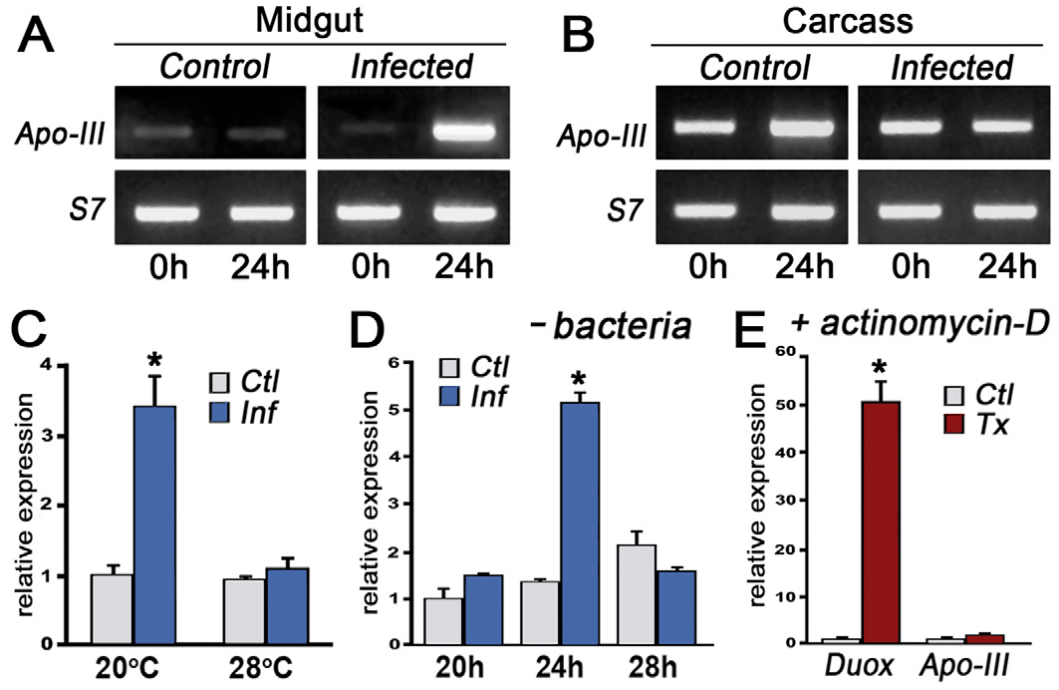
AgApoLp-III expression in response to blood feeding and *Plasmodium berghei* infection in *Anopheles gambiae* (G3 strain) females. (A) AgApoLp-III mRNA expression in midguts of female mosquitoes fed on a healthy (control) or a *P. berghei*-infected mouse. Samples were collected immediately (0 hour) or 24 hours post feeding. Ribosomal S7 protein was amplified as reference. (B) Same as (A) but in carcass samples collected from the same mosquitoes. (C) AgApoLp-III expression in control and infected midguts kept at a temperature permissive for ookinete development (20°C) or at a non-permissive temperature (28°C). (D) Time course of AgApoLp-III induction in female mosquitoes in which gut microbiota have been removed with oral antibiotics. (E) Effect of actinomycin D-induced apoptosis on mRNA expression of dual oxidase (Duox) and AgApoLp-III (Apo-III).

**Figure 4:**
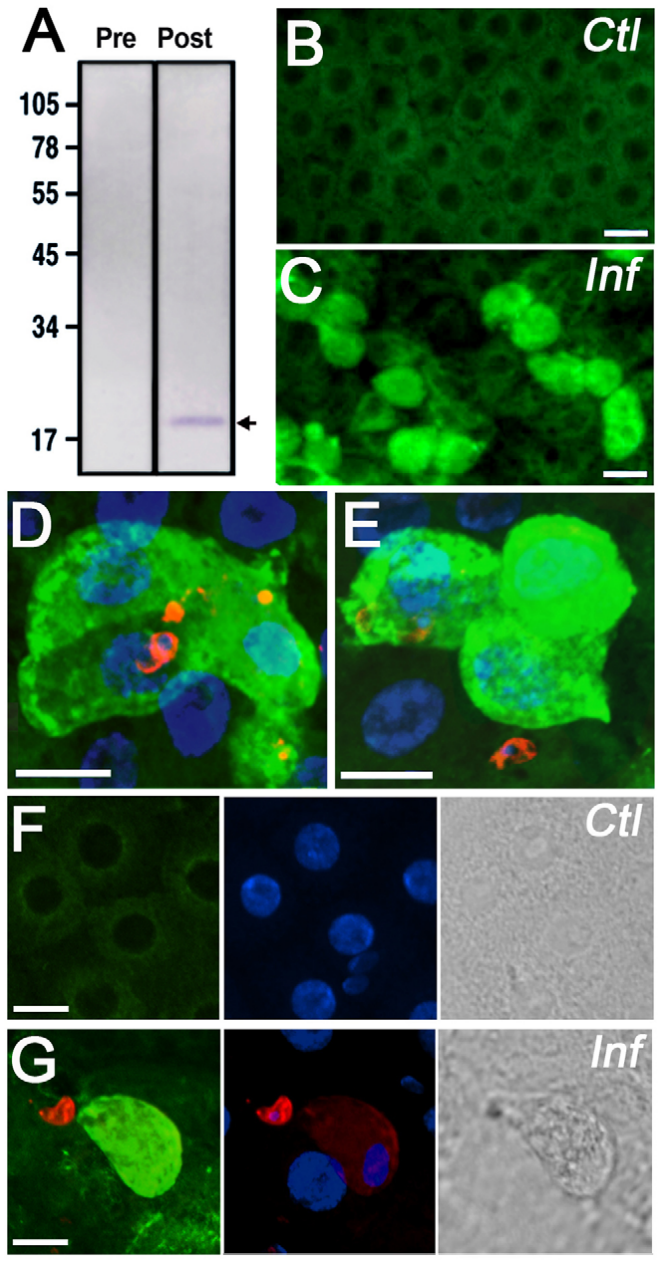
AgApoLp-III protein detection and subcellular localization in control and *Plasmodium*-infected midguts. (A) Western blot analysis of hemolymph from adult females probed with pre-immune or polyclonal anti-AgApoLp-III antiserum. A single band of expected molecular weight (19 kDa) for the mature protein was detected and is indicated by the arrow. (B–G) Subcellular localization of AgApoLp-III (green) in control (B and F) and infected (C, D, E, and G) midguts. The ookinete surface was stained using anti-Pb28 monoclonal antibodies (red) and nuclei with DAPI (blue). Phase contrast images of the midgut surface in control (F) and infected (G) midguts are shown in the right panels.

**Figure 5:**
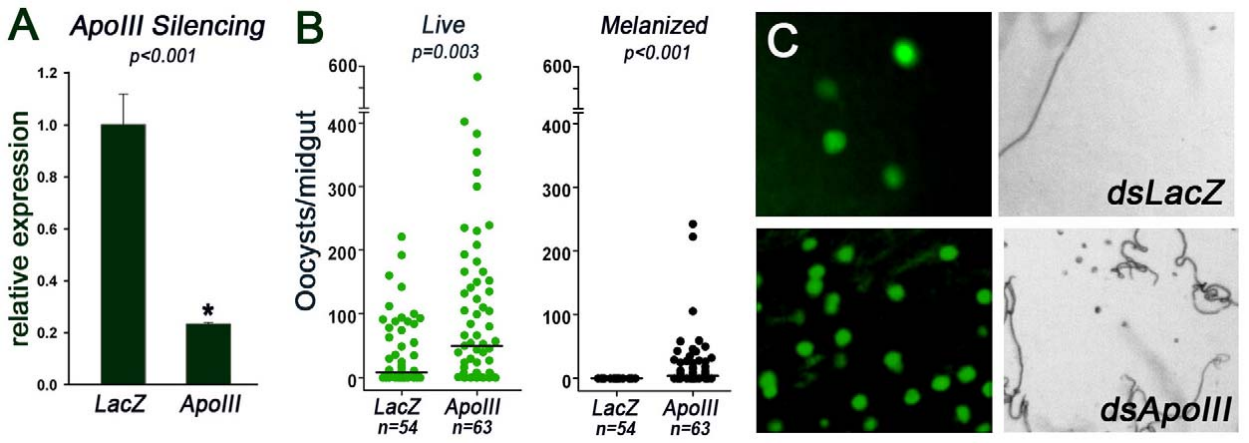
Effect of AgApoLp-III silencing on *Plasmodium berghei* infection in *Anopheles gambiae* (G3 strain) females. (A) Relative abundance of AgApoLp-III mRNA in G3 mosquitoes injected with AgApoLp-III dsRNA (ApoIII) or control LacZ dsRNA (*LacZ*). (B) Effect of AgApoLp-III silencing on the number of live oocysts (green dots; 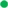) and melanized parasites (black dots; 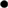) in midguts analyzed seven days post infection. Dots represent the number of parasites present on individual midguts, and the median number of parasites is indicated by the horizontal line. Distributions are compared using the Kolmogorov-Smirnov test; n =  number of mosquitoes, * indicates significant decrease in mRNA levels relative to *dsLacZ*-injected controls. (C) Representative field to illustrate the effect of AgApoLp-III silencing on *Plasmodium* infection 7 days post-feeding. Live oocysts express GFP and exhibit green fluorescence (right panels), while melanized parasites can be observed as dark spots in bright field images (left panels).

A phylogenetic analysis of AgApoLp-III and other known insect members of the apoLp-III family indicates that AgApoLp-III is most similar to ApoLp-IIIs from *Culex quinquefasciatus* (XP_001849278) (59% identity) and *Aedes aegypti* (XP_001659524) (54%) mosquitoes (Figure 2, B and C); however, mosquito apoLp-IIIs are very divergent and only share 19% to 23% aa identity with those of Lepidoptera (Figure 2C). A higher level of sequence conservation is observed between moth sequences such as *H. cunea* (AAQ24031), *Bombyx mori* (AAQ17038.1), *M. sexta* (A29793), *G. mellonella* (CAA07363.1), and *Spodoptera litura* (AAC63377.1) apoLp-IIIs, which share 58% to 72% identity among themselves (Figure 2, B and C).

### Expression of *AgApoLp-III* mRNA in Response to *Plasmodium* Infection

To explore the potential involvement of AgApoLp-III mosquito defense responses to *P. berghei* (murine malaria), AgApoLp-III mRNA expression in the midgut and carcass (the rest of the body without the midgut tissue) was determined in *An. gambiae* G3 females fed on either a healthy control or a *P. berghei*-infected mouse. Samples were collected either immediately (0 hour) or 24 hours after feeding. Feeding on uninfected blood had no effect on AgApo-III expression in the midgut or carcass (Figure 3, A and B); however, AgApoLp-III expression was strongly induced in the midgut of mosquitoes infected with malaria (Figure 3A), while expression in the carcass was not affected (Figure 3B). The increase in AgApoLp-III expression was observed 24 hpi, a time when ookinete midgut invasion is taking place. To investigate whether AgApoLp-III expression was induced in response to ookinetes and rule out a response to some other factor present in the blood of infected mice, two groups of mosquitoes were fed on the same *Plasmodium*-infected mouse and kept either at 20°C (a permissive temperature for *P. berghei* development) or at 28°C, a temperature at which gametocytes die and ookinetes do not form. AgApoLp-III was only induced significantly (P<0.001) when mosquitoes were kept at 20°C (Figure 3C), indicating that the midgut is responding to the presence of ookinetes.

Commensal bacteria are abundant in the gut lumen at the time ookinetes invade midgut epithelial cells [35], [36]. To establish whether AgApoLp-III expression was induced in response to bacteria and/or to *Plasmodium* ookinetes, bacteria were removed by oral administration of antibiotics before mosquitoes were blood fed. AgApoLp-III expression is still induced in *Plasmodium*-infected midguts when the gut microbiota are eliminated (P<0.001) (Figure 3D), indicating that the midgut is responding to the parasite. Transcriptional activation of AgApoLp-III is transient, as it is not observed 20 hpi, is robust at 24 hpi, but is no longer present by 28 hpi (Figure 3D). Together, these data indicate that ookinete invasion induces AgApoLp-III expression in the midgut and could play an important role in the cellular responses to the parasite.

Ookinete invasion causes irreversible cell damage that leads to apoptosis [25] and induces expression of several heme peroxidases [32]. Expression of some of these peroxidases, including the *An. gambiae* dual oxidase (AgDuox) enzyme, also increases when apoptosis is induced in the midgut by oral administration of actinomycin-D [32]. We investigated whether the observed induction of AgApoLp-III following *Plasmodium* infection was related to the apoptotic response of midgut epithelial cells; however, although AgDuox expression is highly induced in midguts treated with actinomycin-D (P<0.001), AgApoLp-III mRNA levels are not affected (Figure 3E).

### Subcellular Localization of AgApoLp-III Protein in *P. berghei*-Infected Midguts

To determine the subcellular localization of AgApoLp-III in *Plasmodium*-infected midguts, recombinant AgApoLp-III protein was expressed in *E. coli* and used to generate polyclonal antibodies. The anti-AgApoLp-III polyclonal serum recognizes a single protein band of the size (19 kDa) predicted for the mature form of AgApoLp-III and that is not detected by the pre-immune serum (Figure 4A). The subcellular localization of AgApoLp-III in the midgut was established by immunofluorescence staining. AgApoLp-III was uniformly expressed at low levels in the cytoplasm of epithelial cells from mosquitoes fed on uninfected mice 24 hours post feeding (Figure 4, B and F); however, in *Plasmodium*-infected midguts, some cells expressed high levels of AgApoLp-III in their cytoplasm (Figure 4C), and ookinetes were observed in close proximity to these cells (Figure 4, D, E, and G). Differential interference contrast images confirmed that the cells expressing high levels of AgApoLp-III protrude toward the midgut lumen (Figure 4G) as part of the apoptotic response triggered by parasite invasion.

### Effect of AgApoLp-III Silencing on *Plasmodium* Infection

AgApoLp-III expression was silenced by systemic injection of dsRNA, and endogenous mRNA levels were reduced by 78% to 90% (Figure 5A). AgApoLp-III silencing increases the median number of live oocysts by more than five fold (P<0.003) (Figure 5B, left panel and 5C), indicating that—when present—ApoLp-III limits *P. berghei* infection. Interestingly, AgApoLp-III silencing also resulted in a moderate increase in the number of melanized parasites during the ookinete-to-oocysts transition (Figure 5B, right panel and 5C).

## Discussion

The high level of divergence between dipteran and lepidopteran apoLp-IIIs sequences may reflect the plasticity of apoLp-IIIs, as divergent amino acid sequences can fold into proteins with similar tertiary structures. Some of the divergence may also be due to adaptation to differences in biology and feeding behavior. For example, adult mosquito females rely on blood as a nutrient source for reproduction, which is very rich in proteins and lipids compared to the flower nectar or fruit juice consumed by adult moths. Mosquito apoLp-IIIs could have diverged as they adapted to meet the functional demands of blood feeding. Notably, we were unable to identify orthologs of mosquito ApoLp-IIIs in any other dipteran insect, including *Drosophila*, precluding a phylogenetic comparison within this order.

In G3 *An. gambiae* females infected with *P. berghei* AgApoLp-III expression was highly induced in response to *P. berghei* infection 24 hpi, when ookinete invasion is taking place (Figure 3, A and D). This induction required the presence of ookinetes, as it was no longer observed when mosquitoes fed on an infected mouse were kept at a temperature that prevents ookinete formation (28°C) (Figure 3C). The induction of AgApoLp-III is not a response to bacteria in the gut lumen, because ookinete invasion still induces expression after the gut microbiota was eliminated by oral administration of antibiotics (Figure 3D); and is not part of a general apoptotic response, because it is not observed when apoptosis of midgut epithelial cells is induced by oral administration of actinomycin D. Interestingly, in the refractory *An. gambiae* L35 strain, AgApoLp-III silencing also greatly increased the number of parasites present 7 days post infection [24]. In L35 females, however, all parasites are still eliminated and melanized in the ookinete-to-oocyst transition [24]. Together, these data indicate that in G3 and L35 mosquitoes AgApoLp-III silencing increases the number of ookinetes that successfully invade the midgut and reach the basal lamina.

The response of *An. gambiae* Yaoundé females to *Plasmodium* infection is very different [24]. In this strain, AgApoLpIII expression 22–25 hours post-feeding was not induced in the midguts of mosquitoes infected with *P. berghei* ANKA 2.34, which generates invasive ookinetes, relative to the control group fed on mice infected with the *P. berghei* ANKA 2.33 strain that lacks gametocytes and does not form ookinetes [24]. In G3 *An. gambiae* females, AgApoLp-III expression in infected midguts is regulated at the transcriptional level. The lack of transcriptional activation of the AgApoLp-III gene in Yaoundé females could explain why silencing AgApoLp-III in this strain had no effect on *Plasmodium* infection [24]. It is surprising that two *An. gambiae* strains considered to be susceptible to *P. berghei* infection (Yaoundé and G3) exhibit such dramatic differences in AgApoLp-III regulation when they are infected with the same parasite. Although the mechanism mediating these differences is unknown, these observations add support to the idea that there is a broad range of compatibility, defined as the extent to which the immune system limits infection, between particular strains of mosquitoes and specific parasites strains [35].

It is not clear why high levels of AgApoLp-III in the cytoplasm of invaded cells are detrimental to the parasite. It is possible that AgApoLp-III could be working as a cytoplasmic pathogen recognition receptor. If this were the case, one would predict that some other cellular responses such as protein nitration may not be triggered effectively when the presence of parasite-derived molecules is not detected.

We have previously shown that ookinete invasion induces high levels of nitric oxide synthase expression in the cell cytoplasm [25] that is followed by peroxidase-mediated protein nitration [32] and proposed that these cellular responses limit *Plasmodium* survival [25] [32]. Studies in vertebrates indicate that apolipoprotein E (apoE), a mammalian homolog of insect apoLp-III, increases nitric oxide production in immune activated RAW cells by increasing arginine uptake [36]. Thus, AgApoLp-III could be enhancing the production of nitric oxide in invaded midgut cells.

AgApoLp-III silencing also promotes *Plasmodium* melanization (Figures 5B and 5C), indicating that, when present, AgApoLp-III inhibits activation of melanization responses in the mosquito. Recently, Seo et al. [37] reported that lipid-bound apoLp-III from *H. cunea* plays a critical role in reducing oxidative stress, whereas this antioxidant activity is absent in the lipid-free state. ApoLp-III in the hemolymph may reduce lipid oxidation and prevent activation of the phenol oxidase cascade.

Our studies revealed that, besides the known roles of ApoLp-IIIs in lipid transport and hemocyte biology, AgApoLp-III expression can be also induced in epithelial cells in response to parasite invasion. Furthermore, expression of AgApoLp-III in the cell cytoplasm enhances antiplasmodial defense. The molecular mechanism mediating these responses is under investigation.
